# Identification of four insertion sites for foreign genes in a pseudorabies virus vector

**DOI:** 10.1186/s12917-021-02887-w

**Published:** 2021-05-12

**Authors:** Chuanjian Zhang, Shiqi Guo, Rongli Guo, Saisai Chen, Yating Zheng, Mengwei Xu, Zhisheng Wang, Yamei Liu, Jichun Wang

**Affiliations:** 1 Institute of Veterinary Immunology and Engineering, National Research Center of Engineering and Technology for Veterinary Biologicals, Jiangsu Key Laboratory for Food Quality and Safety-State Key Laboratory Cultivation Base of the Ministry of Science and Technology, Jiangsu Academy of Agricultural Sciences, 210014 Nanjing, Jiangsu China; 2 Jiangsu Co-innovation Center for Prevention and Control of Important Animal Infectious Diseases and Zoonoses, 225009 Yangzhou, Jiangsu China; 3 College of Veterinary Medicine, Nanjing Agricultural University, 210095 Nanjing, Jiangsu China; 4 Institute of Veterinary Medicine, Jiangsu Academy of Agricultural Sciences, 210014 Nanjing, Jiangsu China

**Keywords:** Pseudorabies virus, Bacterial artificial chromosome, Noncoding region, Insertion site, Spike gene

## Abstract

**Background:**

Pseudorabies virus (PRV) is a preferred vector for recombinant vaccine construction. Previously, we generated a TK&gE-deleted PRV (PRV^Δ^^TK&gE−AH02^) based on a virulent PRV AH02LA strain. It was shown to be safe for 1-day-old piglets with maternal PRV antibodies and 4 ~ 5 week-old PRV antibody negative piglets and provide rapid and 100 % protection in weaned pigs against lethal challenge with the PRV variant strain. It suggests that PRV^TK&gE−AH02^ may be a promising live vaccine vector for construction of recombinant vaccine in pigs. However, insertion site, as a main factor, may affect foreign gene expression.

**Results:**

In this study, we constructed four recombinant PRV-S bacterial artificial chromosomes (BACs) carrying the same spike (S) expression cassette of a variant porcine epidemic diarrhea virus strain in different noncoding regions (UL11-10, UL35-36, UL46-27 or US2-1) from AH02LA BAC with TK, gE and gI deletion. The successful expression of S gene (UL11-10, UL35-36 and UL46-27) in recombinant viruses was confirmed by virus rescue, PCR, real-time PCR and indirect immunofluorescence. We observed higher S gene mRNA expression level in swine testicular cells infected with PRV-S(UL11-10)ΔTK/gE and PRV-S(UL35-36)ΔTK/gE compared to that of PRV-S(UL46-27)ΔTK/gE at 6 h post infection (*P* < 0.05). Moreover, at 12 h post infection, cells infected with PRV-S(UL11-10)ΔTK/gE exhibited higher S gene mRNA expression than those infected with PRV-S(UL35-36)ΔTK/gE (*P* = 0.097) and PRV-S(UL46-27)ΔTK/gE (*P* < 0.05). Recovered vectored mutant PRV-S (UL11-10, UL35-36 and UL46-27) exhibited similar growth kinetics to the parental virus (PRV^Δ^^TK&gE−AH02^).

**Conclusions:**

This study focuses on identification of suitable sites for insertion of foreign genes in PRV genome, which laids a foundation for future development of recombinant PRV vaccines.

## Background

Pseudorabies viruses (PRV) is the causative agent of pseudorabies. Infection with PRV results in high mortality in newborn pigs, respiratory illness in growing pigs, and abortions and stillbirths in sows [1]. The attenuated PRV strains with gE or gE&gI&TK deletion provide higher protection in pigs against the PRV challenge [2, 3]. Recently, we generated a TK&gE-deleted PRV (PRV^Δ^^TK&gE−AH02^), which is safe for 1-day-old piglets with maternal PRV antibodies and 4 ~ 5 week-old PRV antibody negative piglets and can provide complete protection in weaned pigs against challenged with virulent AH02LA strain at 7 days post vaccination [4]. Several previous reports have shown that recombinant PRVs expressing foreign antigens are able to stimulate humoral and cell-mediated immune responses against both PRV and other viruses [5, 6]. Thus, PRV^Δ^^TK&gE−AH02^ might be a technically appropriate vector for the expression of foreign antigens of other swine diseases.

PRV belongs to the herpesvirus group. Its genome is approximately 145kbp containing some nonessential regions that can be replaced by heterologous gene [2]. Insertion of the foreign gene should have a minimal effect on growth characteristics and vector quality of parental virus. Several non-essential genes sites (gG, gI, gE and gI gene, and TK) are usually the targets of foreign gene insertions for PRV vaccine vectors [7–10]. However, insertion of foreign genes into non-essential genes may partly affect properties of the parental virus and expression of foreign antigens [8]. Therefore, it is important to identify other sites in PRV genome where foreign genes can be inserted and expressed stably without disrupting properties of the parental virus. In PRV genome, several noncoding regions are located in the downstream of two open reading frames (ORFs) in the opposite direction [11]. Insertion of a foreign gene into these noncoding regions should not disrupt any viral genes expression. Therefore, noncoding region in the downstream of two ORFs in the opposite direction may be suitable sites for insertion of foreign genes.

In this study, we constructed recombinant PRVs expressing spike (S) gene of a variant porcine epidemic diarrhea virus (PEDV) strain in which S expression cassette was inserted in the noncoding regions (UL11-10, UL35-36 and UL46-27) of PRV^Δ^^TK&gE−AH02^ genome and evaluated the effect of insertion site on antigen expression and growth ability of the vector virus.

## Results

### Construction of PRV-S bacterial artificial chromosome (BAC)

**Fig. 1:**
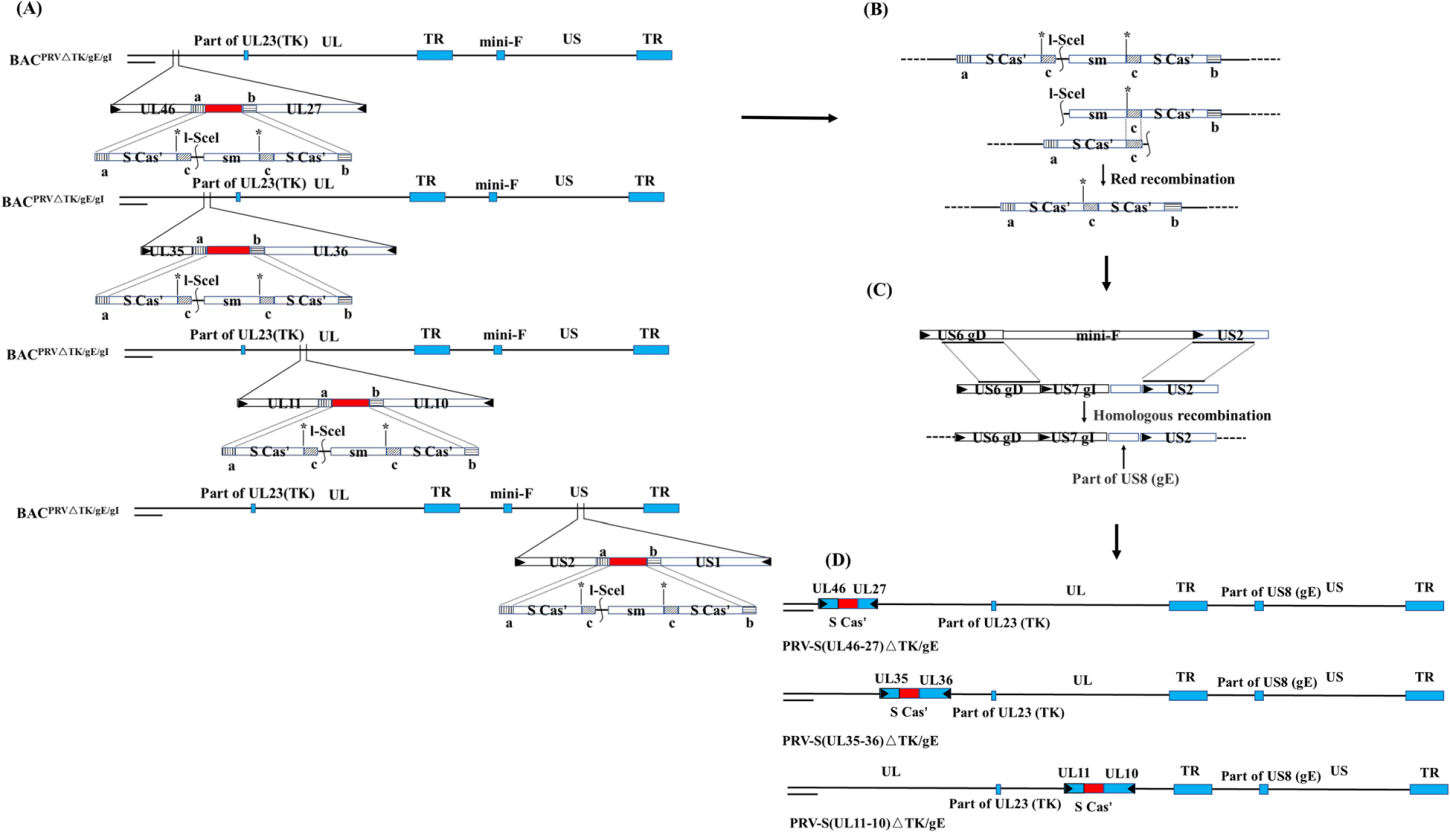
Construction of PRV-S(UL11-10)ΔTK/gE, PRV-S(UL35-36)ΔTK/gE and PRV-S(UL46-27)ΔTK/gE. **a** The S expression cassette with a kanamycin resistance gene was inserted into the noncoding area (UL11-10, UL35-36, UL46-27 or US2-1) through the first recombination to generate four recombinant BAC clones (BAC^PRV−S−KAN(UL11−10)ΔTK/gE/gI^, BAC^PRV−S−KAN(UL35–36)ΔTK/gE/gI^, BAC^PRV−S−KAN(UL46−27)ΔTK/gE/gI^ and BAC^PRV−S−KAN(US2−1)ΔTK/gE/gI^). **b** The second recombination was performed to delete the kanamycin resistance gene and generate the final recombinants (BAC^PRV−S(UL11−10)ΔTK/gE/gI^, BAC^PRV−S(UL35–36)ΔTK/gE/gI^, BAC^PRV−S(UL46−27)ΔTK/gE/gI^ and BAC^PRV−S(US2−1)ΔTK/gE/gI^). **c** Homologous recombination was performed to recover the intact gI gene and part of gE gene (1299 to 1735 bp of gE open reading frame). **d** Schematic presentation of the PRV-S(UL11-10)ΔTK/gE, PRV-S(UL35-36)ΔTK/gE and PRV-S(UL46-27)ΔTK/gE were shown

**Fig. 2:**
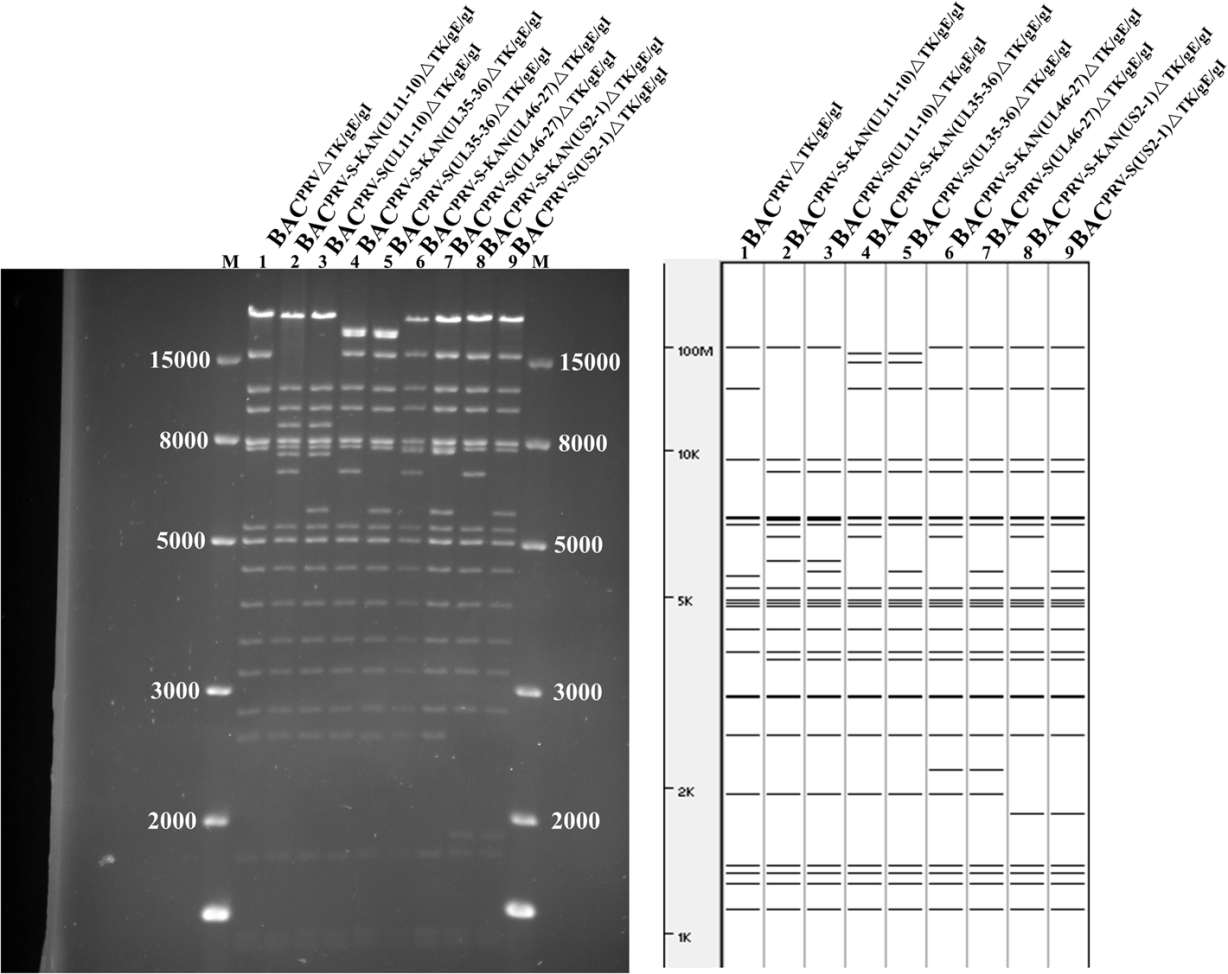
RFLP of BAC^PRVΔTK/gE/gI^, BAC^PRV−S−KAN(UL11−10)ΔTK/gE/gI^, BAC^PRV−S(UL11−10)ΔTK/gE/gI^, BAC^PRV−S−KAN(UL35–36)ΔTK/gE/gI^, BAC^PRV−S(UL35–36)ΔTK/gE/gI^, BAC^PRV−S−KAN(UL46−27)ΔTK/gE/gI^, BAC^PRV−S(UL46−27)ΔTK/gE/gI^, BAC^PRV−S−KAN(US2−1)ΔTK/gE/gI^ and BAC^PRV−S(US2−1)ΔTK/gE/gI^. DNA of BAC^PRVΔTK/gE/gI^, BAC^PRV−S−KAN(UL11−10)ΔTK/gE/gI^, BAC^PRV−S(UL11−10)ΔTK/gE/gI^, BAC^PRV−S−KAN(UL35–36)ΔTK/gE/gI^, BAC^PRV−S(UL35–36)ΔTK/gE/gI^, BAC^PRV−S−KAN(UL46−27)ΔTK/gE/gI^, BAC^PRV−S(UL46−27)ΔTK/gE/gI^, BAC^PRV−S−KAN(US2−1)ΔTK/gE/gI^ and BAC^PRV−S(US2−1)ΔTK/gE/gI^ were digested with *BamH* I. Predicted RFLP pattern with *BamH* I was performed using PRV ZJ01 strain (GenBank: KM061380.1) as a reference

Based on the AH02LA BAC with TK, gE and gI deletion (BAC^PRVΔTK/gE/gI^), noncoding regions (UL11-10, UL35-36, UL46-27 or US2-1) was replaced with S^cas^-KAN (S expression cassette with kanamycin resistance gene) through the first recombination. The kanamycin resistance gene was deleted from the S expression cassette through a second recombination, generating four PRV-S recombinant BACs (BAC^PRV−S(UL11−10)ΔTK/gE/gI^, BAC^PRV−S(UL35–36)ΔTK/gE/gI^, BAC^PRV−S(UL46−27)ΔTK/gE/gI^ and BAC^PRV−S(US2−1)ΔTK/gE/gI^, Fig. 1). Restriction fragment length polymorphism (RFLP) analysis of these constructs with *BamH* I digestion corresponded with the predicted pattern with minor differences (Fig. 2). S expression cassette was confirmed by PCR and sequencing (data not shown).

### Generation of recombinant PRVs expressing S gene of PEDV from cloned DNA

**Fig. 3:**
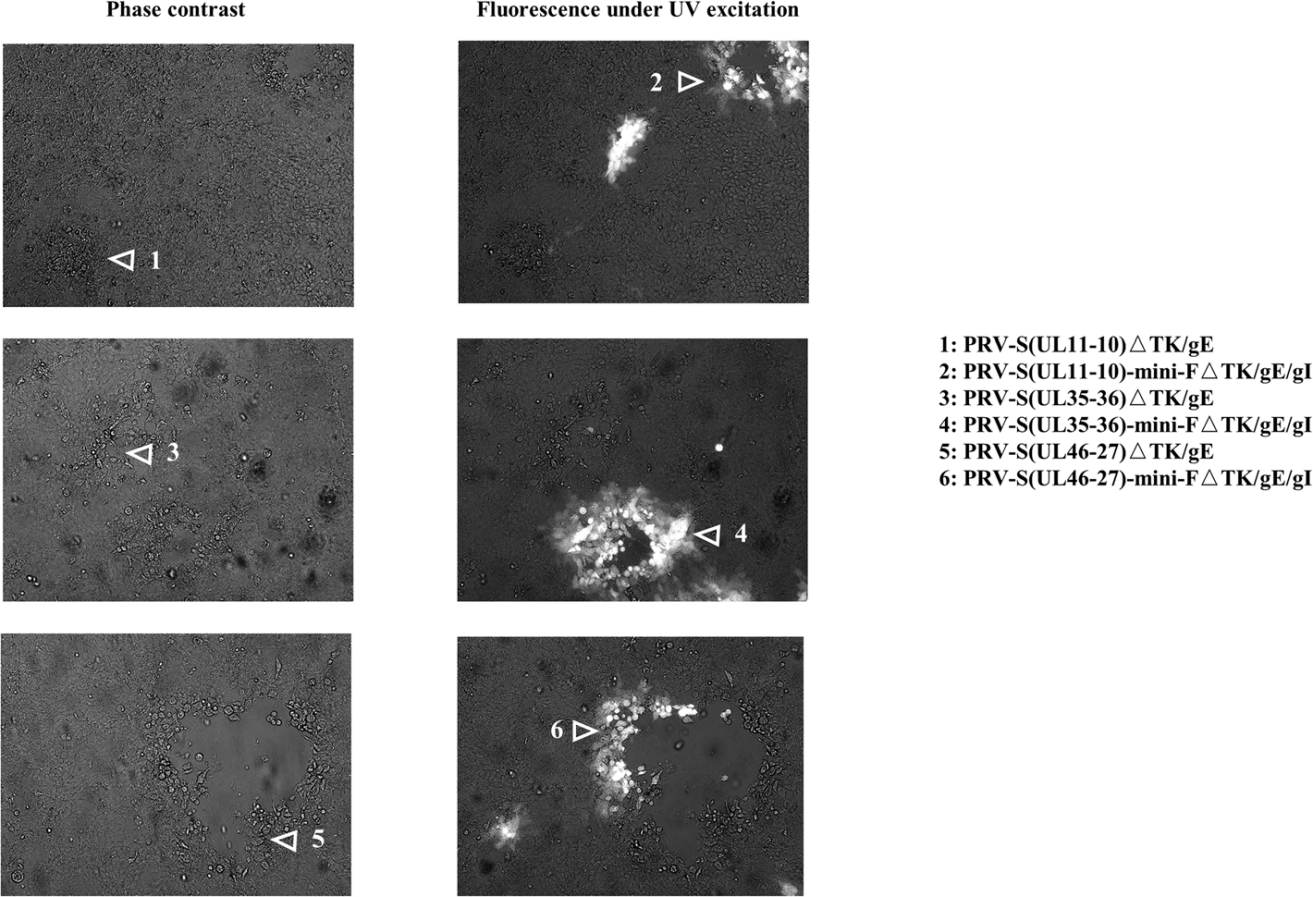
Images of PRV-S(UL11-10)ΔTK/gE, PRV-S(UL11-10)-mini-FΔTK/gE/gI, PRV-S(UL35-36)ΔTK/gE, PRV-S(UL35-36)-mini-FΔTK/gE/gI, PRV-S(UL46-27)ΔTK/gE and PRV-S(UL46-27)-mini-FΔTK/gE/gI under UV excitation and phase contrast are shown. Each panel represents a view of 200 × 200 μm in size

**Fig. 4:**
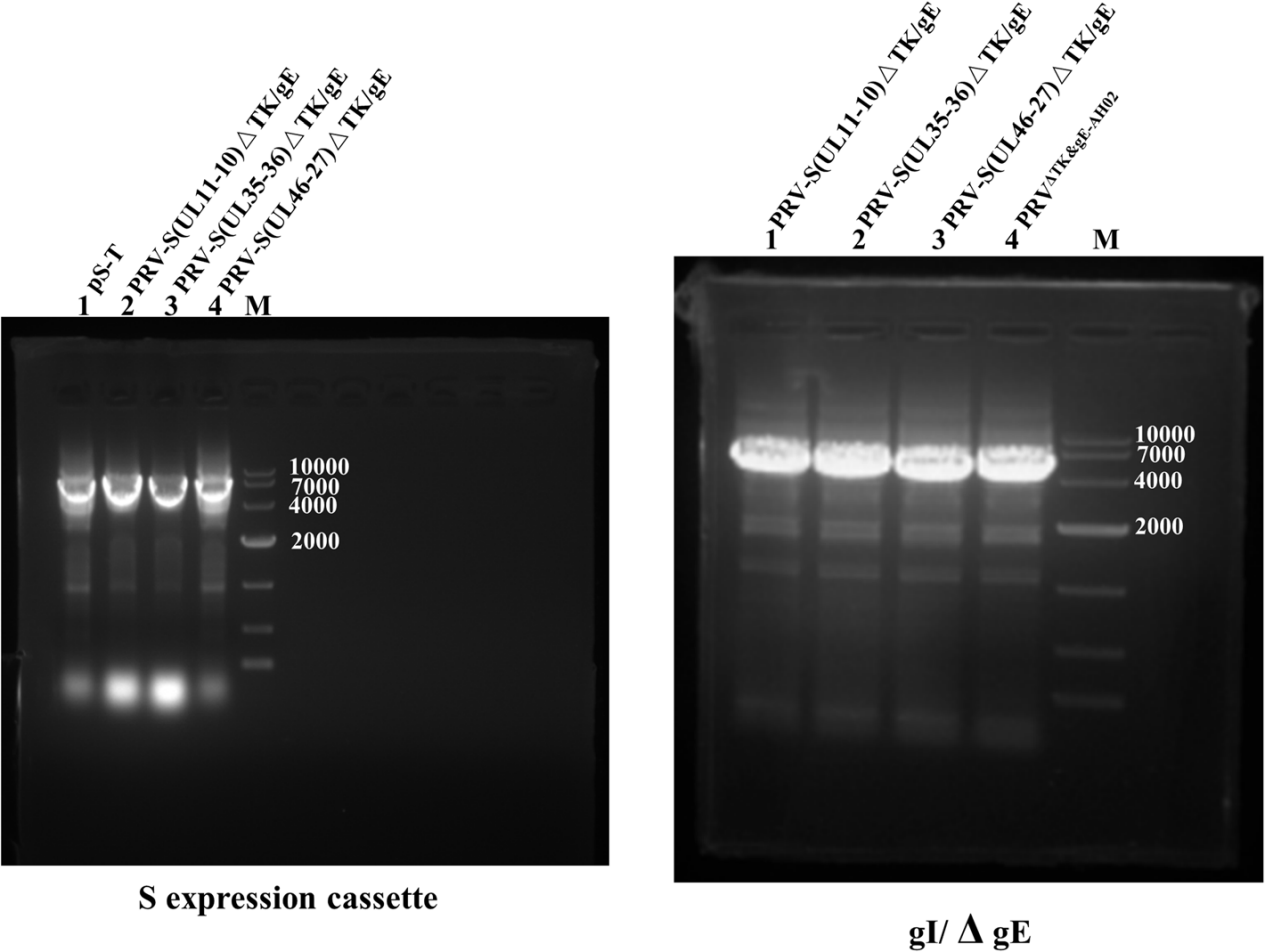
PCR identification of S expression cassette and gI/ΔgE. S expression cassette of PRV-S(UL11-10)ΔTK/gE, PRV-S(UL35-36)ΔTK/gE and PRV-S(UL46-27)ΔTK/gE was identified by PCR with primers (S cas check F/S cas check R), and gI/ΔgE was identified by PCR with primers (PRV ΔgE check F/PRV ΔgE check R)

To generate recombinant PRVs expressing S gene of PEDV, co-transfection of DNA of BAC^PRV−S(UL11−10)ΔTK/gE/gI^, BAC^PRV−S(UL35–36)ΔTK/gE/gI^, BAC^PRV−S(UL46−27)ΔTK/gE/gI^ or BAC^PRV−S(US2−1)ΔTK/gE/gI^ and H1-H2-gI-ΔgE, non-fluorescent plaques of PRV-S(UL11-10)ΔTK/gE, PRV-S(UL35-36)ΔTK/gE and PRV-S(UL46-27)ΔTK/gE were observed under UV light (488nm) (Fig. 3). BAC^PRV−S(US2−1)ΔTK/gE/gI^ failed to rescue the virus, indicating that US2-1 region may be crucial for PRV replication. To obtain a homogeneous population, one plaque was isolated after 5 rounds of plaque purification. The correct sequences of the inserted S expression cassette and H1-H2-gI-ΔgE of PRV-S(UL11-10)ΔTK/gE, PRV-S(UL35-36)ΔTK/gE and PRV-S(UL46-27)ΔTK/gE were confirmed by PCR and sequencing (Fig. 4). To investigate the genetic stability of S expression cassette, the recombinant viruses were passaged 20 times on swine testicular (ST) cells. The viral DNAs were extracted, and S expression cassette was detected by PCR. The correct sequences of the inserted S expression cassette were confirmed by sequencing (data not shown). However, indirect immunofluorescence assay (IFA) and western blotting are further needed to identify S protein expression of the three recombinant viruses passaged 20 times on ST cells.

### Comparative analysis of S expression capacity

**Fig. 5:**
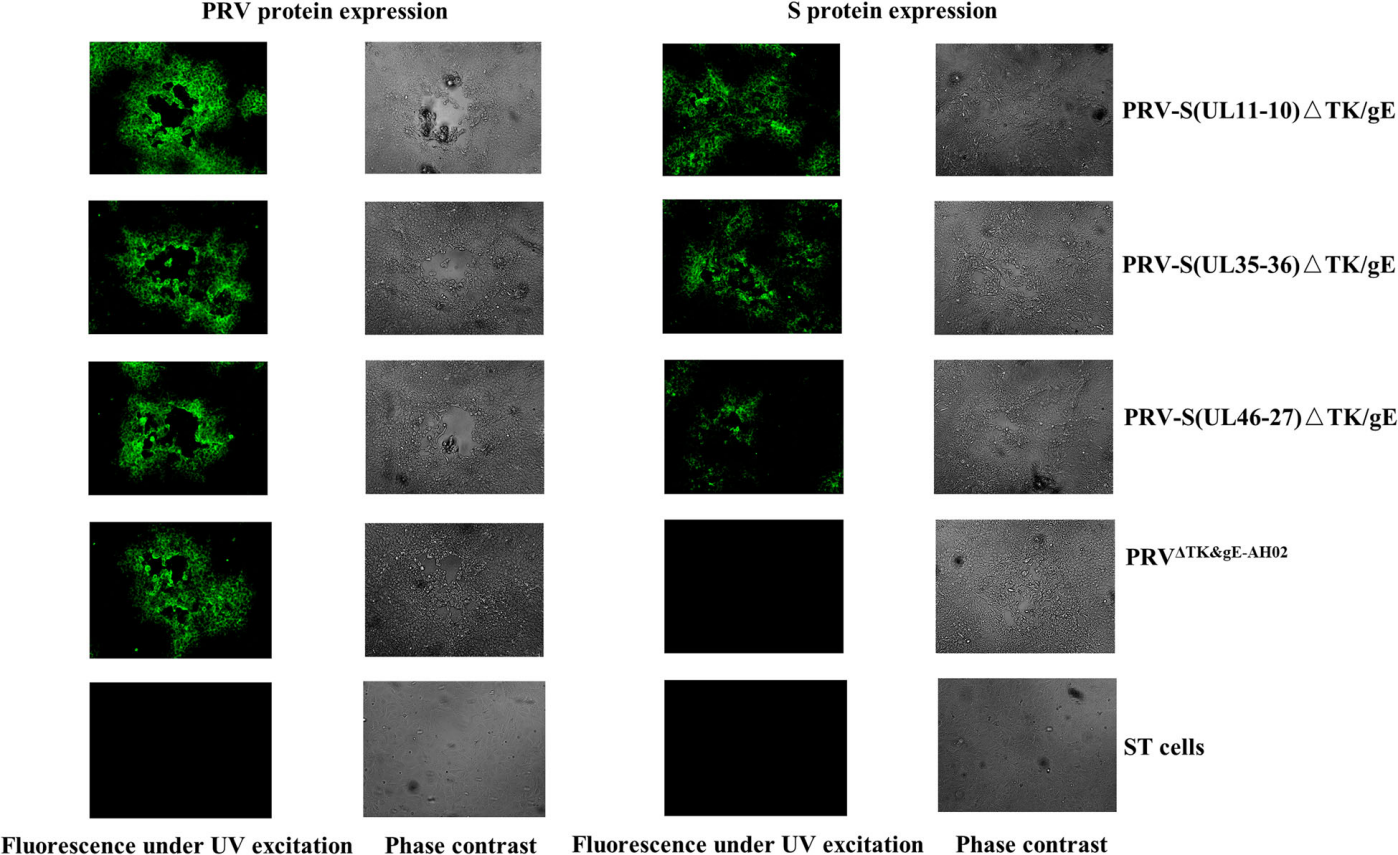
Confirmation of S and PRV protein expression by the recombinant PRV using indirect immunofluorescence assay. Pig anti-sera against PEDV or PRV in conjunction with FITC labelled anti-pig secondary antibodies were employed to verify S and PRV protein expression. Each panel represents a view of 200 × 200 μm in size

**Fig. 6:**
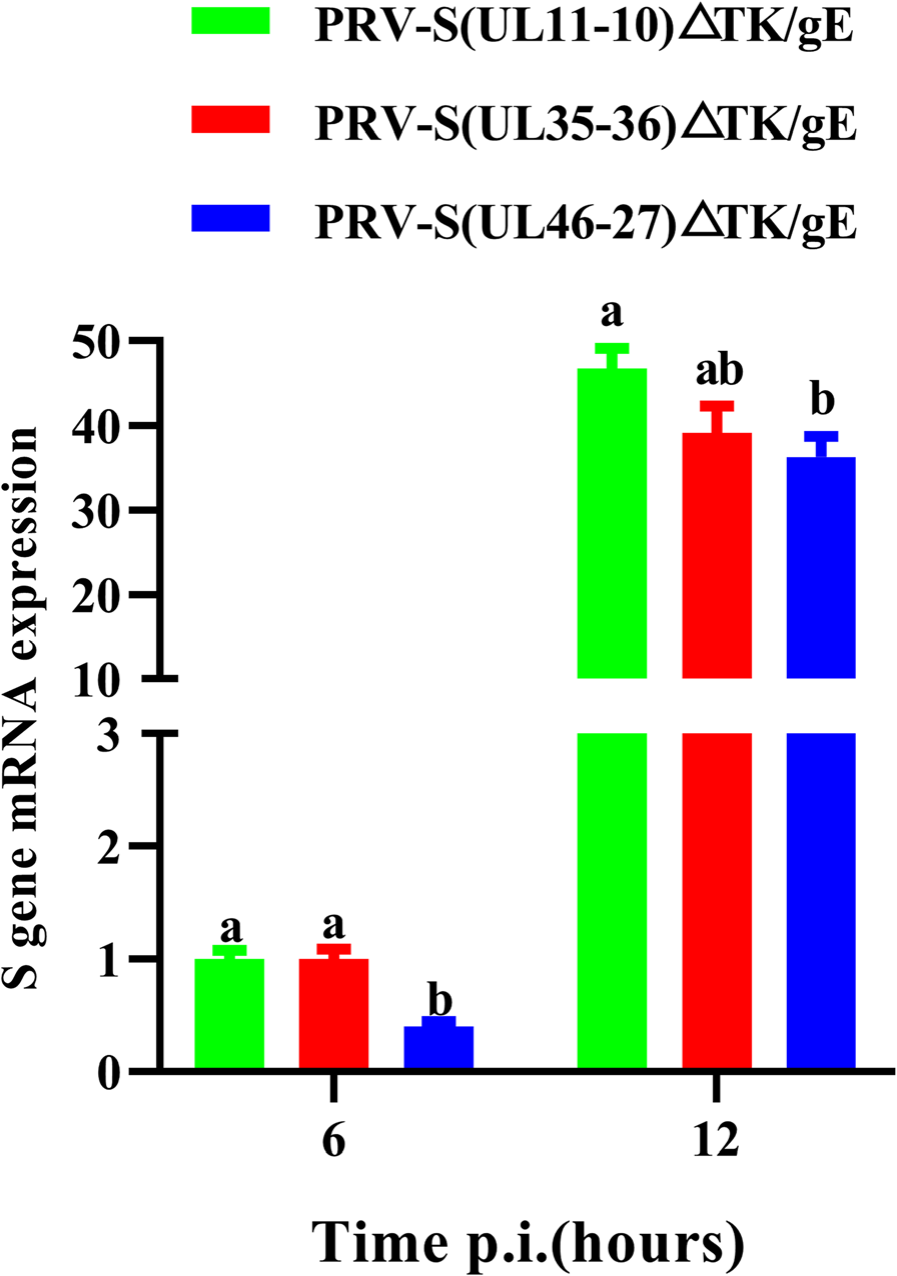
S gene mRNA expression on ST cells infected with PRV-S(UL11-10)ΔTK/gE, PRV-S(UL35-36)ΔTK/gE and PRV-S(UL46-27)ΔTK/gE at a MOI of 10 at 6 and 12 h post infection. Data are expressed in relative arbitrary units, in comparison with the values measured in ST cells infected with PRV-S(UL11-10)ΔTK/gE at 6 h post infection and taken as 1.00. Data were presented as mean ± SEM, and analyzed using one-way ANOVA with a Tukey’s post − hoc test (SPSS Inc., Chicago, IL, USA)

S and PRV protein expression in recombinant PRVs were confirmed by using IFA in infected ST cells (Fig. 5). Cells infected with PRV-S(UL11-10)ΔTK/gE, PRV-S(UL35-36)ΔTK/gE and PRV-S(UL46-27)ΔTK/gE reacted with anti-S and anti-PRV antibody, emitting a green fluorescent signal under UV light (488 nm). However, no fluorescence for S protein was detectable in cells infected with PRV^Δ^^TK&gE−AH02^ or ST cells (Fig. 5). S gene mRNA expression of recombinant PRVs in ST cells was also confirmed by real-time PCR (RT-PCR) in Fig. 6. S gene mRNA expression in three recombinant viruses differed at 6 and 12 h post infection, and cells infected with PRV-S(UL11-10)ΔTK/gE and PRV-S(UL35-36)ΔTK/gE exhibited higher S gene mRNA expression than that infected with PRV-S(UL46-27)ΔTK/gE at 6 h post infection (*P* < 0.05). At 12 h post infection, S gene mRNA expression from PRV-S(UL11-10)ΔTK/gE was higher than those from PRV-S(UL35-36)ΔTK/gE (*P* = 0.097) and PRV-S(UL46-27)ΔTK/gE (*P* < 0.05).

### Growth kinetics of recombinant PRVs expressing S gene of PEDV

**Fig. 7:**
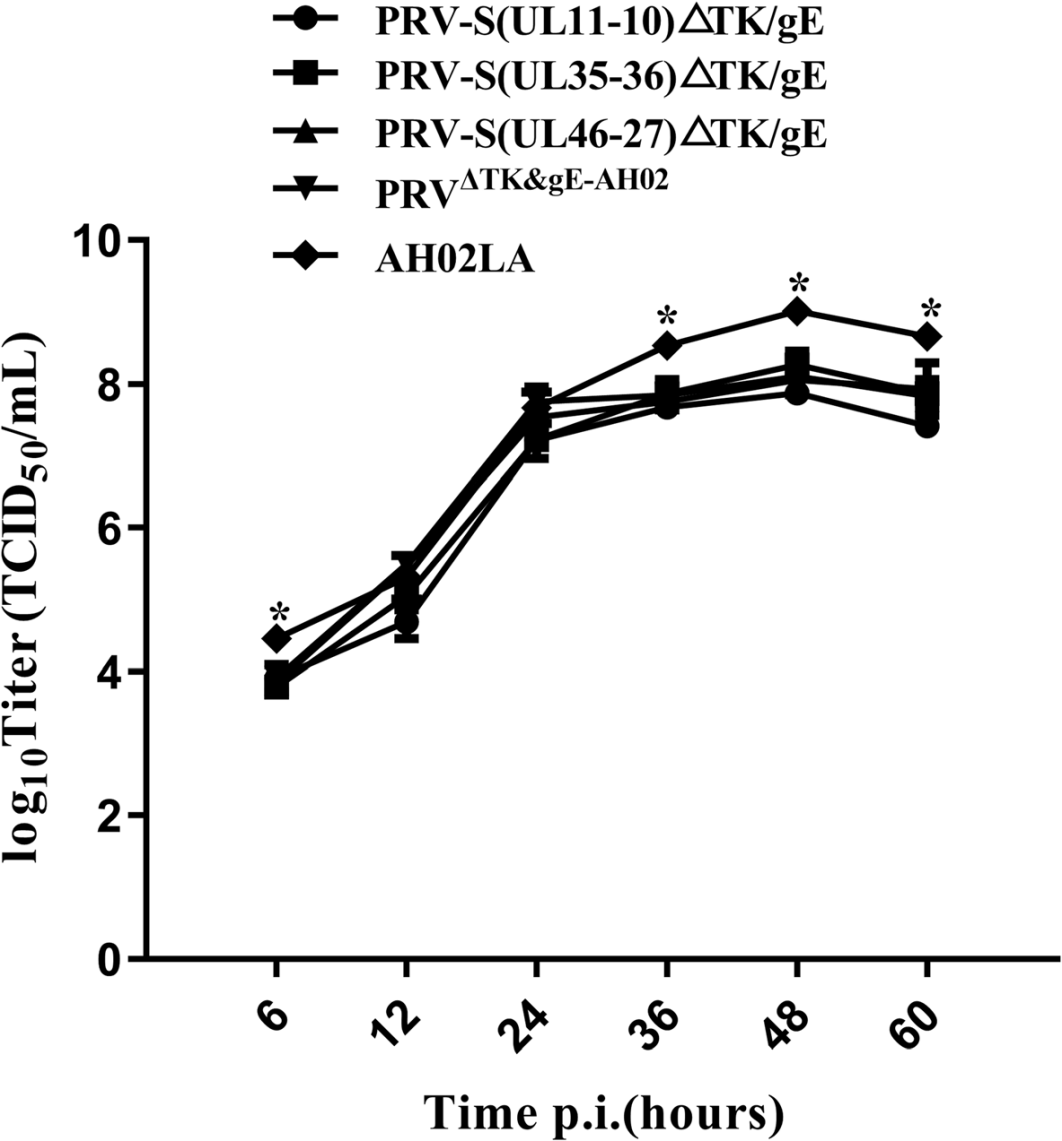
Multi-step growth curves of AH02LA, PRV^ΔTK&gE−AH02^, PRV-S (UL11-10)ΔTK/gE, PRV-S(UL35-36)ΔTK/gE and PRV-S(UL46-27)ΔTK/gE. ST cells were infected with AH02LA and the four mutants at a MOI of 0.01. At 6, 12, 24, 36, 48 and 60 h post infection, the culture cells were harvested and then were titrated in ST cells. Asterisks indicate statistical significance between AH02LA and the four mutants (*p < 0.05). Data were presented as mean ± SEM, and analyzed using one-way ANOVA with a Tukey’s post − hoc test (SPSS Inc., Chicago, IL, USA)

The growth kinetics of the PRV AH02LA, PRV^ΔTK&gE−AH02^, PRV-S(UL11-10)ΔTK/gE, PRV-S(UL35-36)ΔTK/gE and PRV-S(UL46-27)ΔTK/gE on ST cells were shown in Fig. 7. The growth kinetics of PRV-S(UL11-10)ΔTK/gE, PRV-S(UL35-36)ΔTK/gE and PRV-S(UL46-27)ΔTK/gE were similar to those of PRV^ΔTK&gE−AH02^, indicating that S expression cassette insertion did not affect the replication of parental virus. However, at 6, 36, 48 and 60 h post infection, the titers of PRV^ΔTK&gE−AH02^, PRV-S(UL11-10)ΔTK/gE, PRV-S(UL35-36)ΔTK/gE and PRV-S(UL46-27)ΔTK/gE were lower than PRV AH02LA, which may due to the deletion of TK and gE. Peak titers for PRV-S(UL11-10)ΔTK/gE, PRV-S(UL35-36)ΔTK/gE and PRV-S(UL46-27)ΔTK/gE were 10^7.87^, 10^8.27^ and 10^8.05^ TCID_50_/mL, respectively (Fig. 7).

## Discussion

It has been reported that attenuated PRV strain may be developed as virus vectors to express foreign genes [2]. A TK&gE-deleted (PRV^Δ^^TK&gE−AH02^) previously constructed in our lab may be an appropriate vector for the expression of foreign antigens. However, insertion site is the main factor influencing foreign gene expression. In PRV genome, noncoding region in the downstream of two ORFs with opposite direction may be suitable sites for insertion of foreign genes. In this study, we constructed four recombinant PRV-S BACs based on AH02LA BAC with TK, gE and gI deletion in which S expression cassette was inserted in the different noncoding regions (UL11-10, UL35-36, UL46-27 or US2-1). Based on virus rescue, PCR and IFA analysis, S gene inserted in the UL11-10, UL35-36 and UL46-27 region of PRV were successfully expressed. However, BAC^PRV−S(US2−1)ΔTK/gE/gI^ in which US2-1 region was replaced with S expression cassette failed to rescue the recombinant PRV. S gene mRNA expression of PRV-S(UL11-10)ΔTK/gE and PRV-S(UL35-36)ΔTK/gE were significantly higher than that of PRV-S(UL46-27)ΔTK/gE at 6 h post infection. At 12 h post infection, PRV-S(UL11-10)ΔTK/gE exhibited highest S gene mRNA expression among the three recombinant PRVs. The study first reported noncoding region of PRV as foreign gene insertion site, which provides new appropriate sites for construction of PRV-based vaccines.

Compared with traditional homologous recombination, BAC-based genetic manipulation platform for PRV facilitates the generation of recombinant PRVs with gene deletions or insertions. A few of PRV genomes have been maintained in BACs as infectious clones [12, 13]. BAC^PRVΔTK/gE/gI^ was previously constructed in our lab [4], and used to generate PRV-S BAC by *En Passant* protocol in this study. It is surprising for us to find out that a recombinant PRV BAC in which US2-1 region was replaced with S expression cassette was not successfully rescued, which indicates that US2-1 may be crucial for PRV replication.

To develop a PRV vector-based vaccine, it is important that the recombinant virus still retains the growth ability of the vector virus. In this study, there was no difference in virus titers between three recombinant PRVs expressing S gene and the parent virus PRV^Δ^^TK&gE−AH02^, indicating that the insertion of the S gene in the UL11-10, UL35-36 or UL46-27 did not influence the growth of the vector virus. Since three sites used in this study for insertion of S expression cassette were noncoding region in the downstream of two ORFs with opposite direction, the insertion of S expression cassette at the above three sites might not influence the PRV replication on ST cells.

Foreign gene expression level is correlated with live vector vaccine efficacy [14]. Therefore, we investigated S expression of three recombinant PRVs by IFA and RT-PCR. The results revealed that S protein inserted in the UL11-10, UL35-36 and UL46-27 region of PRV were successfully expressed. It is well known that the S protein plays an important role in the induction of neutralizing antibodies and has been used in the preparation of effective vaccines [15, 16]. It suggests that the three recombinant viruses might induce a certain degree of protection against challenge by the virulent strain of PEDV. ST cells infected with PRV-S(UL11-10)ΔTK/gE and PRV-S(UL35-36)ΔTK/gE showed significantly higher S gene mRNA expression level than those infected with PRV-S(UL46-27)ΔTK/gE at 6 h post infection. At 12 h post infection, S gene mRNA expression level on ST cells infected with PRV-S(UL11-10)ΔTK/gE was higher than those of PRV-S(UL35-36)ΔTK/gE and PRV-S(UL46-27)ΔTK/gE. However, western blotting is further needed to validate the S protein expression. Considering S gene expression and viral replication rate observed in this study, we propose that UL11-10 may be an optimal insertion site among three noncoding regions for foreign gene expression. However, due to the different situations in vitro and in vivo, it is worthwhile to detect and compare the expression of the three recombinant viruses in vivo in further studies. A previous study has shown that the viral genome DNA was detected in the brain, heart, lung, liver, kidney, and spleen tissues of PRV-Fa infected mice, but not in PRV-ΔgE/ΔgI/ΔTK infected group at 72 h post infection [6]. When the infection time was extended to 200 h, viral DNA was detected in the brain and lungs of mice infected with PRV-ΔgE/ΔgI/ΔTK. We speculate that the copy number of PRV^ΔTK&gE−AH02^ is lower compared to the PRV AH02LA in vivo. Since the three noncoding regions in the downstream of two ORFs with opposite direction used in this study for insertion of S expression cassette may be nonessential for the replication of PRV, the insertion of S expression cassette at the above three sites might not influence the PRV replication in vivo.

## Conclusions

We constructed three recombinant PRVs in which S expression cassette was inserted in the UL11-10, UL35-36 or UL46-27 region of the PRV^Δ^^TK&gE−AH02^ genome through BAC technology using homologous recombination. S gene inserted in three noncoding regions of PRV was expressed. High S gene mRNA expression level was discovered in ST cells infected with PRV-S(UL11-10)ΔTK/gE at 6 and 12 h post infection among the tested recombinant PRVs. Furthermore, S expression cassette inserted in the UL11-10, UL35-36 or UL46-27 region did not affect the replication of parental virus. The identification and comparison of the insertion sites in PRV genome in this study will be useful for the further development of recombinant PRV vaccines. Future studies involving animal experiments are necessary to evaluate safety and efficacy of the three recombinant PRVs.

## Methods

### Viruses, cells and plasmids

The PRV AH02LA strain (a PRV variant) was isolated and identified in our lab [17]. A gE/TK-deleted PRV (PRV^Δ^^TK&gE−AH02^) based on the PRV AH02LA strain were constructed in our lab [4]. ST cells from CVCC was cultured in Dulbecco’s Modified Eagle’s Medium (Gibco, USA) supplemented with penicillin (100U/mL), streptomycin (100 µg/mL) and 2 ~ 10 % fetal calf serum in a humidified incubator with 37 °C and 5 % CO_2_. The S gene was amplified by reverse transcription PCR with a pair of primers (S-F and S-R) from the jejunal tissue sample of a dead piglet that was diagnosed as PEDV-positive using reverse transcription PCR as previously described [18]. The amplified fragment was cloned to downstream of a pMCMV promoter. The resulting S gene expression cassette was inserted pMD19-T (Takara), and named pS-T. Furthermore, to construct plasmid pS-KAN-T, a kanamycin resistance gene was cloned into the *EcoR* I site in pS-T.

### Bacterial manipulations, PCR and sequencing

AH02LA BAC with TK, gE and gI deletion (BAC^PRVΔTK/gE/gI^) constructed previously in our lab [4], was used to generate PRV-S BACs. Electroporation was carried out as described earlier [19]. Plasmid and PRV-S BAC DNA were performed with commercial kits (QIAGEN) according to manufacturer’s instructions. PRV-S BAC was confirmed by RFLP with *BamH* I.

Primers KAN ins S F/KAN ins S R (Table 1) with two *EcoR* I restriction sites in both terminals for cutting and ligation were used to insert a kanamycin resistance gene into plasmid S-T. Primers (PRV ins S cas UL11-10 F/R, PRV ins S cas UL35-36 F/R, PRV ins S cas UL46-27 F/R or PRV ins S cas US2-1 F/R; Table 1) were used to insert the S^cas^-KAN into the UL11-10, UL35-36, UL46-27 or US2-1 of BAC^PRVΔTK/gE/gI^ through the *En Passant* protocol [20]. Specific primers (S cas check F/R, Table 1) were used to verify the sequence of the inserted S gene expression cassette. A pair of primers (PRV BAC H1 F and PRV BAC H2 R) were used to amplify a DNA fragment (H1-H2-gI-ΔgE) including the gI gene, part of gE with the deletion of the 1286 bp fragment (position 13 to 1298), and upstream and downstream homologous sequences using PRV LA-A^B^ strain [17] DNA as template. The primers PRV ΔgE check F and PRV ΔgE check R were used for sequencing gI and ΔgE gene. S expression cassette were confirmed by sequencing (S cas check F and S cas check R).

**Table 1:** Primers for PCR, sequencing or RT-PCR

Primer	Sequence (5’- 3’)
S-F	ATGAAGTCTTTAACCTACTTCTGG
S-R	TCACTGCACGTGGACCTTTT
Kan ins S F	CCGGAATTCAAACGCCATGTACTTTCCCACCATTGACGTCAATGGGCTAGGATGACGACGATAAGTAGGGATAAC
Kan ins S R	GGCGAATTCGGGTAATGCCAGTGTTACAACCA
PRV ins S cas UL11-10 F	TCGCGGGCGTACTGACTGCAATAAACCCGTTTGTCATACTCTAGTGGATCCCCCAACTCC
PRV ins S cas UL11-10 R	CGGCGACGAGGTCGTGTACGAGAACCTCGGCTTTGAATAATTGTCGACTCTAGAGGATCCG
PRV ins S cas UL35-36 F	TGCCCTCGCCCCTCGCCCTAGCCCCGCGCGATCAATAAAGCTAGTGGATCCCCCAACTCC
PRV ins S cas UL35-36 R	GTGGACCTATTTCAGGTCCGCCTGATTCTTGGTTAATAAATTGTCGACTCTAGAGGATCCG
PRV ins S cas UL46-27 F	TGCCCCCCTGTGTGGAAATAAAGTTTTTTTCTAATTCTGTCTAGTGGATCCCCCAACTCC
PRV ins S cas UL46-27 R	CTACCAGCGCCTCGAGAACGAGGACCCCGACGCCCCCTAGTTGTCGACTCTAGAGGATCCG
PRV ins S cas US2-1 F	CAACGGACGCGAGCGCGCCCCGCGATGTACCATCTCCTAGCTAGTGGATCCCCCAACTCC
PRV ins S cas US2-1 R	CTCTGTTGTGCCCTCAATAAACACGGCGGCCCGCCGCTCGTTGTCGACTCTAGAGGATCCG
PRV BAC H1 F	GTACCCGTACACCGAGTCGT
PRV BAC H2 R	TTGTGGACCCGCGCGAACAT
S cas check F	CTAGTGGATCCCCCAACTCC
S cas check R	TTGTCGACTCTAGAGGATCC
PRV ΔgE check F	AGCCCCGGGAAGATAGCCAT
PRV ΔgE check R	ATCGCGGAACCAGACGTCGAAG
S exp F	CGGTAACACTAATGCTACTGCGCG
S exp R	CGATCATTATCCCATGTTATGCCG
Beta actin F	AGAGCGCAAGTACTCCGTGT
Beta actin R	ACATCTGCTGGAAGGTGGAC

### Generation of recombinant PRVs expressing S gene of a PEDV variant

S expression cassette was inserted into the noncoding area of BAC^PRVΔTK/gE/gI^ to replace nucleotide fragments of UL11-10, UL35-36, UL46-27 or US2-1 through the *En Passant* method [20]. Briefly, S^cas^-KAN with 40 bp homologous sequences of PRV in both terminals were amplified. After digestion with *Dpn* I, four PCR products was electroporated into GS1783 with BAC^PRVΔTK/gE/gI^ to achieve the first recombination at the UL11-10, UL35-36, UL46-27 or US2-1 sites. Four target recombinant clones were generated by deletion of the kanamycin resistance gene through the second recombination (Fig. 1). Selected clones were confirmed by RFLP after digestion with *BamH* I, and named BAC^PRV−S(UL11−10)ΔTK/gE/gI^, BAC^PRV−S(UL35–36)ΔTK/gE/gI^, BAC^PRV−S(UL46−27)ΔTK/gE/gI^ and BAC^PRV−S(US2−1)ΔTK/gE/gI^. Moreover, S gene expression cassette was confirmed using PCR and sequencing. To obtain recombinant PRVs expressing S gene of PEDV, ST cells were transfected with approximately 1 µg BAC^PRV−S(UL11−10)ΔTK/gE/gI^, BAC^PRV−S(UL35–36)ΔTK/gE/gI^, BAC^PRV−S(UL46−27)ΔTK/gE/gI^ or BAC^PRV−S(US2−1)ΔTK/gE/gI^ and 1 µg H1-H2-gI-ΔgE. One to two days after transfection, non-fluorescent plaques were selected and purified to obtain homogeneous population of recombinant PRVs expressing S gene of PEDV under UV light (488nm). The S expression cassette and H1-H2-gI-ΔgE were identified with PCR and sequencing. To evaluate the genetic stability of S gene, the recombinant viruses were passaged 20 times on ST cells, and S expression cassette was verified using PCR and sequencing.

### Indirect immunofluorescence assay

ST cells were infected with PRV^Δ^^TK&gE−AH02^ or recombinant PRVs expressing S gene of PEDV. At 2 day post inoculation, cells were washed 3 times with phosphate buffered saline (PBS) and fixed with cold fixing solution (96 % ethanol: acetone = 3:1) for 20 min. Cells were then washed three times with PBS and blocked with PBS + 10 % bovine serum albumin for 1 h. After this blocking reaction, the cells were incubated with anti-S or anti-PRV pig serum produced in our lab at 37℃ for 1 h. After washing three times with PBS, the cells were incubated with FITC-labeled goat anti-pigs IgG (Solarbio, diluted 1:100) at 37 °C for 1 h. Cells were then washed as above and analyzed by inversion fluorescence microscope.

### Real‐time PCR

To evaluate S gene mRNA expression on the cell surface, ST cells in the six-well plates were infected with PRV^ΔTK&gE−AH02^ or recombinant PRVs expressing S gene of PEDV at a multiplicity of infection (MOI) of 10. At 6 and 12 h post infection, infected-cells were separately harvested. Total RNA of infected-cells was extracted using TRIzol reagent [21]. A total of 1 µg total RNA from different treatments was reverse transcribed using a PrimeScript® RT Reagent Kit with gDNA Eraser (Takara Bio). RT-PCR with a pair of primers for S gene (S exp F/R, Table 1) was carried out on Roche Light Cycler® 480 system (Roche Diagnostics, Burgess Hill, UK) using SYBR Premix Ex Taq dye (Takara) [22]. Each cDNA was analyzed in triplicate, and sample data were normalized to Beta actin expression using the 2^−ΔΔCt^ method.

### Multi‐step growth kinetics

Multi-step growth kinetics of PRV AH02LA, PRV^ΔTK&gE−AH02^ and recombinant PRVs expressing S gene of PEDV were tested on ST cells with a MOI of 0.01 as described previously [13], the culture cells were harvested at 6, 12, 24, 36, 48 and 60 h post infection, and then were titrated in cell monolayers. Experiments were performed in triplicate.

## Data Availability

The datasets used and analyzed during the current study are available from the corresponding author on reasonable request.

## Abbreviations

BAC: Bacterial artificial chromosome
gE: Glycoprotein E
gG: Glycoprotein G
gI: Glycoprotein I
IFA: Indirect immunofluorescence assay
MOI: Multiplicity of infection
PBS: Phosphate buffered saline
PED: Porcine epidemic diarrhea
PEDV: Porcine epidemic diarrhea virus
ORFs: Open reading frames
PRV: Pseudorabies virus
RFLP: Restriction fragment length polymorphism
RT-PCR: Real-time PCR
S: Spike
ST cells: Swine testicular cells
TCID_50_: 50% tissue cultureinfectious dose
TK: Thymidine kinase
UL: Unique long
US: Unique short

## References

[1] Mettenleiter TC (2000). Aujeszky’s disease (pseudorabies) virus: the virus and molecular pathogenesis–state of the art, June 1999. Vet Res.

[2] Dong B, Zarlenga DS, Ren X: An overview of live attenuated recombinant pseudorabies viruses for use as novel vaccines. J Immunol Res. 2014;2014:824630. 10.1155/2014/824630 PMC4068083 24995348

[3] Zhang C, Guo L, Jia X, Wang T, Wang J, Sun Z (2015). Construction of a triple gene-deleted Chinese Pseudorabies virus variant and its efficacy study as a vaccine candidate on suckling piglets. Vaccine.

[4] Wang J, Song Z, Ge A, Guo R, Qiao Y, Xu M (2018). Safety and immunogenicity of an attenuated Chinese pseudorabies variant by dual deletion of TK&gE genes. BMC Vet Res.

[5] Tong W, Zheng H, Li GX, Gao F, Shan TL, Zhou YJ (2020). Recombinant pseudorabies virus expressing E2 of classical swine fever virus (CSFV) protects against both virulent pseudorabies virus and CSFV. Antiviral Res..

[6] Feng Z, Chen J, Liang W, Chen W, Li Z, Chen Q, Cai S (2020). The recombinant pseudorabies virus expressing African swine fever virus CD2v protein is safe and effective in mice. Virol J.

[7] Hubner A, Keil GM, Kabuuka T, Mettenleiter TC, Fuchs W (2018). Efficient transgene insertion in a pseudorabies virus vector by CRISPR/Cas9 and marker rescue-enforced recombination. J Virol Methods.

[8] Katharina K, Elke L, Teifke JP, Mettenleiter TC, Walter F (2014). Immunization of pigs with an attenuated pseudorabies virus recombinant expressing the haemagglutinin of pandemic swine origin H1N1 influenza A virus. J Gen Virol..

[9] Peeters B, Bienkowska-Szewczyk K, Hulst M, Gielkens A, Kimman T (1997). Biologically safe, non-transmissible pseudorabies virus vector vaccine protects pigs against both Aujeszky’s disease and classical swine fever. J Gen Virol.

[10] Tian ZJ, Zhou GH, Zheng BL, Qiu HJ, Ni JQ, Yang HL (2006). A recombinant pseudorabies virus encoding the HA gene from H3N2 subtype swine influenza virus protects mice from virulent challenge. Vet Immunol Immunopathol.

[11] Pomeranz LE, Reynolds AE, Hengartner CJ (2005). Molecular biology of pseudorabies virus: impact on neurovirology and veterinary medicine. Microbiol Mol Biol Rev..

[12] Gu Z, Jing D, Wang J, Hou C, Sun H, Yang W (2015). A novel inactivated gE/gI deleted pseudorabies virus (PRV) vaccine completely protects pigs from an emerged variant PRV challenge. Virus Res..

[13] Smith GA, Enquist LW (1999). Construction and transposon mutagenesis in *Escherichia coli* of a full-length infectious clone of pseudorabies virus, an alphaherpesvirus. J Virol..

[14] Li K, Liu Y, Liu C, Gao L, Zhang Y, Gao Y (2016). Effects of different promoters on the protective efficacy of recombinant Marek’s disease virus type 1 expressing the VP2 gene of infectious bursal disease virus. Vaccine..

[15] Bosch BJ, van der Zee R, de Haan CA, Rottier PJ (2003). The coronavirus spike protein is a class I virus fusion protein: structural and functional characterization of the fusion core complex. J Virol..

[16] Cruz DJ, Kim CJ, Shin HJ (2008). The GPRLQPY motif located at the carboxy-terminal of the spike protein induces antibodies that neutralize porcine epidemic diarrhea virus. Virus Res.

[17] Wang J, Guo R, Qiao Y, Xu M, Wang Z, Liu Y (2016). An inactivated gE-deleted pseudorabies vaccine provides complete clinical protection and reduces virus shedding against challenge by a Chinese pseudorabies variant. BMC Vet Res..

[18] Fan B, Yu Z, Pang F, Xu X, Zhang B, Guo R et al: Characterization of a pathogenic full-length cDNA clone of a virulent porcine epidemic diarrhea virus strain AH2012/12 in China. Virology. 2017;500:50–61. 10.1016/j.virol.2016.10.011 PMC7111662 27770703

[19] Wang J, Osterrieder N (2011). Generation of an infectious clone of duck enteritis virus (DEV) and of a vectored DEV expressing hemagglutinin of H5N1 avian influenza virus. Virus Res.

[20] Tischer BK, Smith GA, Osterrieder N (2010). En passant mutagenesis: a two step markerless red recombination system. Methods Mol Biol.

[21] Chomczynski P, Sacchi N (1987). Single-step method of RNA isolation by acid guanidinium thiocyanate-phenol-chloroform extraction. Anal Biochem.

[22] Zhang C, Liu Y, Chen S, Qiao Y, Zheng Y, Xu M et al: Effects of intranasal pseudorabies virus AH02LA infection on microbial community and immune status in the ileum and colon of piglets. Viruses. 2019;11(6):518. 10.3390/v11060518 PMC6631256 31195631

